# Two-Step Approach Using Degradable Magnesium to Inhibit Surface Biofilm and Subsequently Kill Planktonic Bacteria

**DOI:** 10.3390/biomedicines9111677

**Published:** 2021-11-12

**Authors:** Pei-Chun Wong, Ren-Yi Wang, Long-Sheng Lu, Wei-Ru Wang, Jason Shian-Ching Jang, Jia-Lin Wu, Tai-Yuan Su, Ling-Hua Chang

**Affiliations:** 1 Department of Orthopedics, School of Medicine, College of Medicine, Taipei Medical University, Taipei 11031, Taiwan; pcwong0424@tmu.edu.tw; 2 Department of Orthopedics, Taipei Medical University Hospital, Taipei 11031, Taiwan; 3 Orthopedics Research Center, Taipei Medical University Hospital, Taipei 11031, Taiwan; 4 Graduate Institute of Biomedical Materials and Tissue Engineering, College of Biomedical Engineering, Taipei Medical University, Taipei 11031, Taiwan; 144012@h.tmu.edu.tw (R.-Y.W.); lslu@tmu.edu.tw (L.-S.L.); 5 Department of Radiation Oncology, Taipei Medical University Hospital, Taipei Medical University, Taipei 11031, Taiwan; 6 School of Biomedical Engineering, College of Biomedical Engineering, Taipei Medical University, Taipei 11031, Taiwan; amber16743@tmu.edu.tw; 7 Department of Mechanical Engineering, National Central University, Taoyuan 32001, Taiwan; 8 Institute of Materials Science and Engineering, National Central University, Taoyuan 32001, Taiwan; 9 Centers for Regional Anesthesia and Pain Medicine, Wan Fang Hospital, Taipei Medical University, Taipei 11600, Taiwan; 10 Department Electrical Engineering, Yuan-Ze University, Chung-Li 32003, Taiwan

**Keywords:** bacterial infection, magnesium, biodegradation, spalling, biofilm, alkaline, antibacterial

## Abstract

Bacterial infection remains a great risk in medical implantation surgery. In this paper, we found that degradable metals may be a feasible alternative option of antibacterial implantation materials. It is known that the spalling mechanism of magnesium (Mg) during degradation leads to Mg ions-induced alkaline environment, which is harmful to planktonic bacteria. In this study, we showed that alkaline pH environment is almost harmless to those adhesive bacteria protected in well-formed biofilms. Moreover, experimental results demonstrated that the biofilm formed in the place where Mg spalls are destroyed, releasing the covered bacteria to be planktonic in the alkaline environment. As a result, the colonization of biofilms continues to shrink during the degradation of Mg. It implies that if degradable metal is employed as implantation material, even if bacterial infection occurs, it may be possibly cured without second surgery.

## 1. Introduction

The entry of pathogenic bacteria which leads to periprosthetic infection are mainly by three routes: (1) direct implantation, (2) hematogenic infection, and (3) reactivation of latent infection [1]. Bacteria such as coagulase-negative *staphylococci* (30–43%), *staphylococcus aureus* (12–23%), *streptococci* (9–10%), *enterococci* (3–7%), Gram-negative organisms (3–6%), and anaerobes (2–4%) can cause periprosthetic infection [2].

The cytoplasmic pH value of bacteria must be steadily maintained to provide structural integrity for cytoplasmic proteins to grow [3]. Most bacteria can tolerantly survive and grow under environments of pH value 5.5–9.0, while their cytoplasmic pH lies in the narrow range of 7.4–7.8, due to the compensation relative to the external milieu by self-acidification or self-alkalization [4]. The adapting mechanisms include increasing metabolic acid, increasing ATP synthase, changes in the cell surface properties, and increasing expression and activity of monovalent cation or proton antiporters. There is also evidence that monovalent cation and proton antiporters can stabilize the alkaline pH for many bacteria [5,6,7,8,9]. Satio et al. has proposed that the activities of enzymes would stabilize the pH value of neutral cytoplasm when bacteria are under extreme alkaline conditions [10].

Biofilm, also called a city of microbes, can be defined as a sessile microbial consortium established in a 3D structure. There are four stages of biofilm formation: (1) cell attachment: the planktonic bacteria begin to contact and moreover to attach to the surface of object; (2) microcolony formation: the attached bacteria proliferate, forming the monolayer and further secreting an extracellular matrix that contains extracellular polymeric substances (EPS), protein, extracellular DNA (eDNA), and polysaccharides (this stage is known as primary biofilm); (3) biofilm maturation: the monolayer further forms multilayers and the biofilm develops to be of a mushroom-shaped structure that contains heterologous populations; and (4) biofilm dispersion: the biofilm disperses the bacteria living inside for further colonization [11,12]. The four stages of biofilm formation periodically result in an expansion of bacterial colonization, accelerating intensively the process of infection, especially when the biofilm in stage three dominates.

The biofilms consist of multicellular prokaryotic and/or eukaryotic communities which are embedded in a matrix composed, at least partially, of the microbial community [13]. The compositions of biofilm are mainly extracellular polymeric substances (EPS), carbohydrate-binding proteins, fimbriae, flagella, other adhesion fibers, and eDNA. EPS is the primary matrix material of the biofilm, and accounts for over 50% of the total organic carbon of biofilm [14]. The composition and structure of the polysaccharides determine the primary conformation of biofilm [15]. The antimicrobial resistance properties of biofilm are primarily due to EPS [16]. The well-formed biofilms cause most precarious infections in orthopedic and dental implants due to their super resistance, up to 1000-fold or more so than planktonic bacteria, to conventional antibiotics and the body’s immune system [17,18,19]. These studies revealed anecdotally that it was particularly difficult to attack the bacteria covered by the well-formed biofilm. However, there exists no direct evidence.

So far, there is no effective medical treatment to cure infection after implant surgery. Once the infection occurs, normally, surgeons consider removing the infected implant, prescribing antibiotics, and then placing a new implant. Therefore, the patients may suffer from the second surgery, which also requires medical resources. Cell attachment of the bacteria on the material surface is the first step in biofilm formation. Multiple reports have studied surface modification or drug delivery methods to avoid bacterial attachment on surfaces and to kill the bacteria [20,21,22,23]. Antibacterial property to resist initial cell attachment of metallic implant materials, such as stainless steel, Ti, and Ti6Al4V alloy, can be improved by functional coating [20,21]. Various alloy compositions have been used to control an adverse environment for initial bacterial attachment on surfaces [24,25,26]. Many researchers have reported that nano/micro scale surface topographies present antiadhesion properties or harmful contact to bacteria [27,28,29].

However, besides the mentioned studies, which are mainly dedicated to the prevention of bacterial attachment by developing either medicines or materials, there exists few valid non-surgery treatments to account for bacterial infection, which generally occurs if the biofilm in stage three has developed. Magnesium (Mg) and its alloys have been widely investigated and developed for implant applications due to their degradability, Young’s modulus (similar to bones), and osteoinductivity [30,31]. The degradable and unstable Mg surface spalls out, and generates corrosion products during degradation [32,33,34,35]. The crack on the surface is initially generated from grain boundaries [36,37]. Several studies have indicated that the released corrosion products from degraded Mg cause an increase in the local pH value, which is harmful to planktonic bacteria [30,38,39]. Moreover, Mg ions released from biodegradable Mg play an important role in orthopedic surgery, due to their ability to enhance new bone formation and antibacterial properties for infection control [38,40,41,42,43,44,45].

Characklis et al. reported that the level of microbial colonization appears to be more active, with a rougher surface due to the diminished shear force [46]. Rough surface topographies can promote bacterial adhesion, but this can also be affected by the shape and size of bacteria, and the interactions between bacteria and material surfaces [47,48,49,50,51,52]. In this study, we used the same-shaped metals which were all manufactured to the similar roughness.

Lee et al. reported that cell adhesion and growth is strong on surfaces with water contact angles of 40°–70° [53]. Microorganisms attach to hydrophobic and nonpolar surfaces more easily than hydrophilic materials [54,55,56]. These results were, at times, contradictory, due to a lack of standardized methods to analyze surface hydrophobicity. Certainly, a hydrophobic interaction occurs between bacteria and the surface to resist initial bacterial cell attachment; once the bacteria endure the repulsive forces of the substrate, it attaches irreversibly [15]. In our experiment, we also confirmed that a hydrophobic surface has stronger resistance to bacterial attachment than a hydrophilic surface.

**Figure 1:**
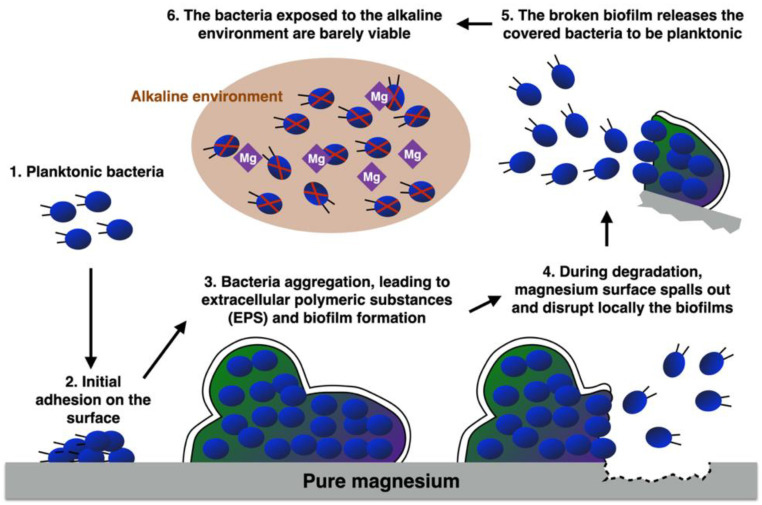
Schematic reaction of bacterial biofilm formation to the degradable Mg.

In this study, we hypothesized and then confirmed the antibacterial mechanism of degradable metals as an implant material (see Figure 1 for demonstration). Planktonic bacteria attach to the alien degradable metal, and develop to stage 2–4. Degradable metal spalls out, releases positive ions, and breaks the well-formed biofilms, so that the bacteria are released to be planktonic. These planktonic bacteria become barely viable in the alkaline environment, thus further bacterial attachment can be prevented. It is a novel break-and-attack two-step mechanism which damages the structure of biofilm, and further destroys the released (planktonic) bacteria in the alkaline environment, compressing the colonization of biofilms. Therefore, if using degradable Mg as an implant material, even if bacterial infection occurs, it can be possibly cured from such a two-step mechanism, so that the patient may avoid a second surgery.

## 2. Materials and Methods

### 2.1. Study Design

We designed experiments to compare the formations of biofilm on various metallic surfaces. The volumetric change of biofilm and the percentage of biofilms in different stages were observed. In the experiment, we used three groups, including degradable Mg substrate (Mg group), nondegradable titanium (Ti) substrate (Ti group), and Ti substrate accompanied by Mg (Ti^+Mg^ group). Between the first two groups, there were two different factors: alkaline environment (Mg group) versus neutral environment (Ti group), and degradable surface (Mg group) versus nondegradable surface (Ti group). The comparisons between the two can be compensated by the Ti^+Mg^ group, which has an alkaline environment and a nondegradable surface.

We also evaluated the viability of planktonic bacteria for the five groups: Mg, Ti, Ti^+Mg^, hydrogen peroxide (H_2_O_2_, 10 M, 10 μL in each well, positive control), and phosphate-buffered saline (PBS, 1×, 10 μL in each well, negative control).

### 2.2. Sample Preparation and Grouping

Pure Mg (>99.9% purity) and pure Ti (>99.9% purity) were obtained from a supplier (Gredmann Group, Taiwan) and machined into cuboids with dimensions of 6 mm × 6 mm × 3 mm. The samples were then polished on all surfaces by 800-grit sandpapers manually, using the same procedure to ensure similar roughness and profiles.

### 2.3. Bacterial Biofilm Culture

DH5-alpha *Escherichia coli* (*E. coli*) was chosen for immersion tests on the metallic substrates, since it forms biofilms easily and it is biosafe. Bacterial suspensions were prepared and cultured as follows. A single colony was selected from the pre-culture on an LB agar plate and was then dropped into liquid LB broth in a falcon tube. An *E. coli* colony was selected and incubated in 3 mL of LB broth for 6 h at 37 °C with shaking mode. Bacterial suspension and LB broth were mixed at a ratio of 1:3. Then, 1 mL of the bacterial suspension mixture was added into every well of a 48-well plate, where the 48 wells were grouped into a Ti group, an Mg group, and a Ti^+Mg^ group, and the immersion of biofilms began. For the Mg and Ti groups, the cuboid was pre-placed horizontally into the well. For the Ti^+Mg^ group, the Ti cuboid was placed horizontally for biofilm formation and the Mg cuboid was placed vertically beside it to release Mg ions within the well. During the six hours of incubation, the environment was controlled at temperature of 37 °C.

### 2.4. Wettability Test

The wettability of the surfaces of Mg and Ti were respectively evaluated via contact angle testing after 1, 2, 4, and 6 h of immersion. After washing all the implant surfaces with 99.5% ethanol, and then drying them in an oven (to avoid corrosion process), the wettability test began when a droplet of DI water and LB broth fluid was deposited using a sessile drop method. The image of the droplet and material were captured by camera, and the contact angle was analyzed by Image J software.

### 2.5. Degradation Behavior Investigation—PH Value, Weight Change, and Surface Composition

The degradation behavior of pure Mg and pure Ti cuboids during immersion in LB broth were investigated, as per our previous work [30]. After different immersion periods (1, 2, 4, and 6 h), the pH values of the LB broth were measured by a pH meter (PH500, CLEAN, Taiwan). Moreover, the cuboids were removed from the LB broth, rinsed with chromic acid (180 g/L) to remove corrosion products, rinsed with DI water, and dried in an oven at 100 °C, before weight measurement by an electronic balance (WAGA ANALITYCZNA AS 220.R2, Radwag Wagi Elektroniczne, Poland). Subsequently, the surface composition of samples was analyzed by energy dispersive spectroscopy (EDS) (EDAX Apollo, EDAX, CA, USA). All tests were conducted five times to ensure repeatability.

### 2.6. Biofilm Quantification

The region of biofilms can be identified with the aid of crystal violet staining, by which the composition of biofilms that are required for the initiation of biofilm formation, such as flagella, pili, adhesins, and enzymes (which are involved in cyclic-di-GMP binding for metabolism), and the genes (which are involved in extracellular polysaccharide production), can be visualized [57]. Biofilm formed after various culture periods (0, 1, 2, 4, and 6 h) was fixed with 4% formalin, stained with crystal violet, and then the bound dye was eluted with dimethyl sulfoxide (DMSO). The enzyme-linked immune-sorbent assay reader (SPECTRA MAX 190, MOLECULAR DEVICE, US) captured the image with an optical density of 590 nm. The volume of the dyed biofilms can thus be calculated.

### 2.7. Morphological Observation of Biofilm

The biofilm formed by DH5-alpha *E. coli* after different periods of immersion on different materials was visualized through scanning electron microscopy (SEM) (SU3500; Hitachi, Tokyo, Japan). Before observation, fixation and dehydration processes were carried out to prepare samples for SEM.

### 2.8. Biofilm-Stage-Identification by an Artificial Intelligence (AI) System

**Figure 2:**
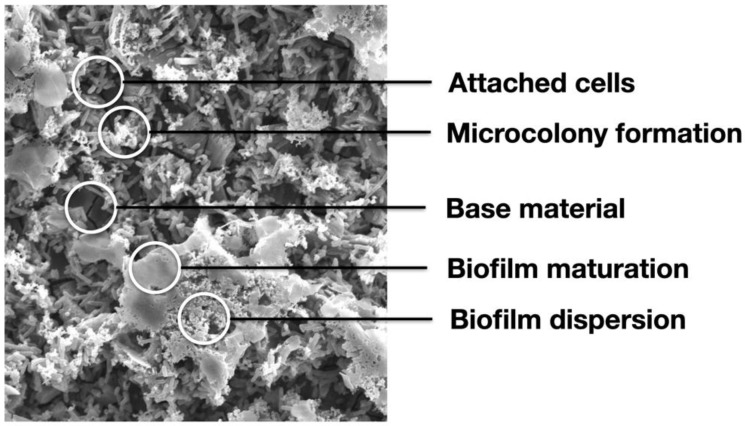
Demonstration of our defined five classes of biofilm: base material, attached cell, microcolony formation, biofilm maturation, and biofilm dispersion.

In this study, a deep convolutional neural network (CNN)-based artificial intelligence system was used to quantify the percentage of biofilm in different stages. The deep CNN model has been proposed to detect biofilm in different stages in our previous report [58]. A total of six SEM images, where the biofilms in different stages are distributed, were used to train the CNN model. Each image measures 1280 × 960 pixels. We manually selected regions of interests (ROI) measuring 40 × 40 pixels, each of which was categorized to five populations, including base material (BM), attached cell (biofilm in stage one), microcolony formation (biofilm in stage two), biofilm maturation (biofilm in stage three), and biofilm dispersion (biofilm in stage four). The shapes of these five populations can be found in Figure 2. Random image shifting and rotation was performed on the training image patches to artificially increase the number of training samples, and thus prevent over-fitting [59]. Particularly, for every selected ROI, three rotations, with respect to the center at random angles, created three other ROIs with the same ROI size. We labeled the five populations, respectively, for the Mg, Ti, and Ti^+Mg^ group. The sample size of each class is shown in Table 1. Using approximately 90% of the collected images, the CNN model was trained with 30 epochs and a batch size of 30. Approximately 10% of the collected images were used for testing the performance of the trained model.

The percentages of biofilm in different stages were detected and calculated by the trained AI system. For the biofilms with respect to the four stages, we also calculated the corresponding volumetric percentage.

### 2.9. Viability of Planktonic Bacteria by MTT Assay

The viabilities of the planktonic bacteria that were not attached to the substrates of the Mg, Ti, and Ti^+Mg^ groups were evaluated by MTT assay. In this experiment, PBS and H_2_O_2_ were the positive control and negative controls, respectively. The bacterial suspension was collected for 100 μL after different periods (1, 2, 4, and 6 h) of immersion, and placed into a well. Then, 10 μL of MTT (3-(4,5-dimethylthiazol-2-yl)-2,5-diphenyltetrazolium bromide) solution (Invitrogen) was added carefully into the well for a further 3 h of incubation. Only viable cells consumed dyed formazan crystals, which was then eluted by DMSO (100 μL). An enzyme-linked immune-sorbent assay reader (Multiskan FC; Waltham, MA, USA) captured the images with an optical density of 560 nm.

### 2.10. Bacterial DNA Damage Detection

One of the strongest pieces of evidence confirming the non-natural apoptosis of the cell is the characterization of DNA damage by comet assay. Sample preparation and alkaline comet assay were performed, according to the protocol provided by the manufacturer (Comet assay kit; Trevigen; Gaithersburg, MD, USA). The cell suspensions were harvested through centrifugation. Cells were then mixed with LMAgarose; 50 μL of this solution was then pipetted onto the CometSlide^TM^. After gelation, the CometSlide^TM^ was immersed into a lysis solution at 4 °C overnight. The lysis solution was then replaced by an alkaline unwinding solution at 4 °C in the dark for 1 h. The comet images were captured by fluorescence microscopy after electrophoresis and SYBR Gold staining.

### 2.11. Statistical Analysis

All results in this study are presented as mean ± standard deviation, and all statistical analyses were performed by SPSS 20. One-way ANOVA tests followed by post hoc Tukey tests were used to analyze all data, with the exception of the results of the contact angle. Contact angle results were analyzed using an independent sample *t* test to examine the differences between materials. The statistical significance was set at a *p*-value smaller than 0.05.

## 3. Results

### 3.1. Wettability of Metallic Materials

**Figure 3:**
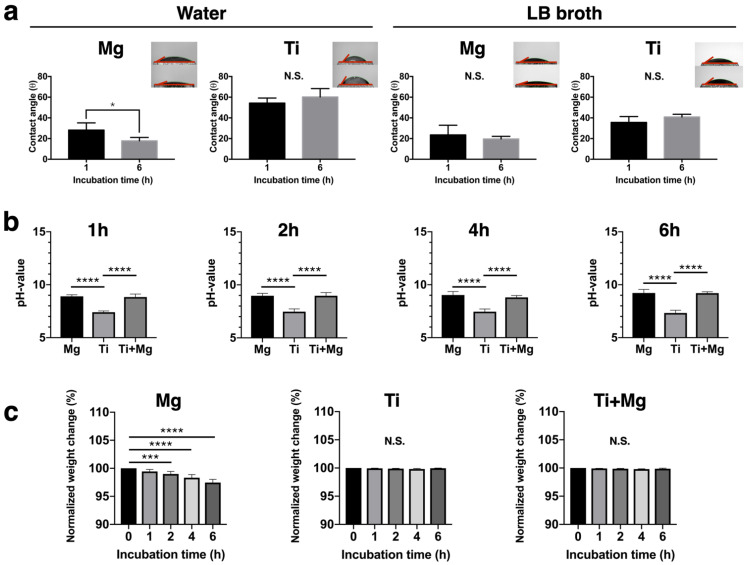
Surface wettability and degradation behavior of Mg and Ti materials. (**a**) Surface wettability of Mg and Ti after 1 and 6 h of incubation in water and LB broth, respectively. (**b**) pH values of the LB broth, with respect to Mg, Ti, and Ti^+Mg^ groups after different incubation periods, and (**c**) weight change of materials at different periods after incubation (*n* = 5 per group; N.S. no significant difference, * *p* < 0.05, *** *p* < 0.005, and **** *p* < 0.001).

The contact angle test can reflect the surface wettability. The contact angle of Mg and Ti in water and LB broth after 1- and 6-h of incubation are recorded in Figure 3a. In the water droplet group, the contact angles after 1 and 6 h for Mg were 28.2° ± 7.0° and 17.8° ± 3.3°, respectively, whereas the contact angle during the same incubation periods for Ti were 54.3° ± 4.9° and 60.2° ± 8.1°. On the other hand, in LB broth, the contact angle of Mg at 1- and 6-h incubation was 23.6° and 9.3°, respectively, while that of Ti at 1- and 6-h incubation was 35.6° ± 5.7° and 40.9° ± 2.6°, respectively. In either the water group or the LB group, Ti is much more hydrophobic than Mg, implying that the surface of Ti has better anti-bacterial-attachment property.

### 3.2. Degradation Behavior—PH Value, Weight Change, and Surface Composition

At the moment of immersion, the pH values for all groups were all 7. As shown in Figure 3b, within one hour of immersion, the pH value of LB broth for the Mg group and Ti^+Mg^ group rapidly increased to 8.90 ± 0.15 and 8.83 ± 0.29, respectively; the pH values for the Mg group and Ti^+Mg^ group increased, respectively, to 9.22 ± 0.33 and 9.21 ± 0.13 after 6 h of immersion. This implies that Mg had degraded within one hour of immersion. On the contrast, the pH value of Ti group remained around 7 after immersion.

As shown in Figure 3c, there are no significant weight changes of the Ti substrates in either the Ti or the Ti^+Mg^ groups. The weight in the Mg group decreased to near 98% after 6 h of incubation, which confirmed Mg had slightly degraded.

Table 2 shows that the surface composition respectively at 1, 2, 4, and 6 h after immersion for the three groups. The Ti samples have no composition change at each time point in both the Ti group and the Ti^+Mg^ group. In contrast, the compositions of Na, P, and Cl were detected on the Mg surface; these compositions were all matched with that of LB broth, which indicates that the Mg samples interacted with the LB broth during immersion.

### 3.3. Morphology of Biofilm

**Figure 4:**
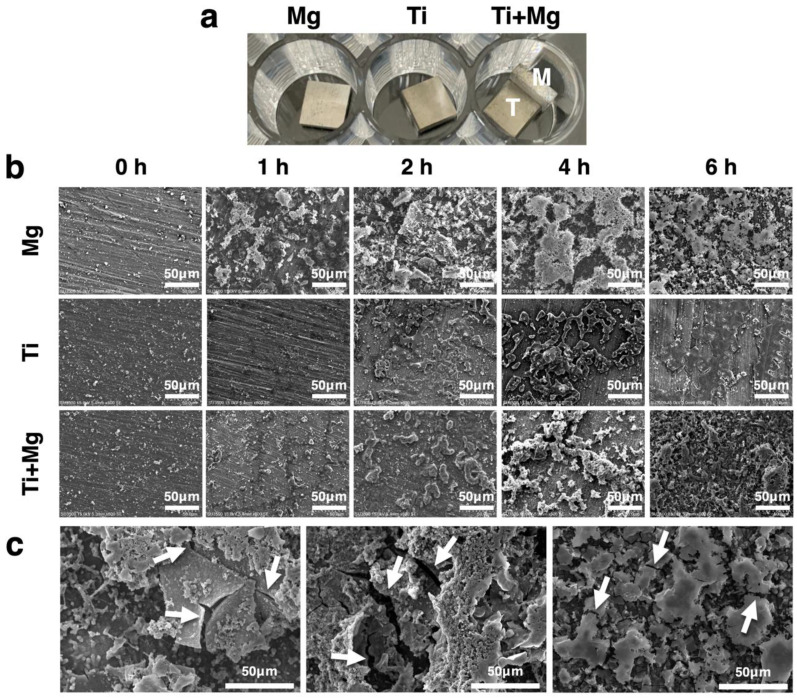
(**a**) Material configuration of Mg, Ti, and Ti^+Mg^ groups for the culture of DH5-alpha *E. coli* culture. (**b**) SEM images of the surface of Mg group, Ti group, and Ti^+Mg^ group at 0, 1, 2, 4, and 6 h of incubation. (**c**) The disrupted biofilms and the crack of Mg surface at six hours of incubation (indicated by arrow).

The SEM image in Figure 4b shows the surface morphology of biofilms. Within one hour of culture, bacteria had already attached and begun to form biofilm on the substrates for all groups. The SEM images of the Mg group showed that within one hour after culture, due to degradation of Mg, there appeared to be a tectonic plate-like surface, and the nearby biofilms were broken. The biofilms continued to be disrupted more severely. After six hours of incubation, a crack on the surface of Mg appeared, and the locally surrounding biofilms were destroyed (Figure 4c). For the Ti group, the territory of biofilm slightly expanded during the six hours of culture. The biofilm dispersed in the Ti group after four hours of incubation, which is a result of the growth cycle. The biofilm damage for the Mg group was much more severe, indicating that the broken biofilms in the Mg group may have been caused by factors other than the natural growth cycle.

Moreover, it can be seen from Figure 4b that the bacterial cell has difficulty to initially attach to the surface of Ti, which coincides with the result in Section 3.1. Nevertheless, we will show that once a biofilm has grown mature on the surface of Ti, it is unlikely to be eradicated; on the other hand, though Mg has weaker resistance to initial bacterial attachment, its degradation mechanism leads to the disruption of the well-formed biofilms, implying that infection can be possibly cured merely with the Mg ions induced in an alkaline environment, in conjunction with some medical treatment.

### 3.4. The Confusion Matrix of Testing the CNN Model

As seen in Table 3, a confusion matrix demonstrates discrepancies between actual class labels and predicted classification results. The rows correspond to the actual class labels while the columns correspond to the detection results made by the CNN model. Thus, the diagonal elements show the number of correct classifications made for each class and the off-diagonal elements show the number of errors made.

### 3.5. Biofilm Quantification

**Figure 5:**
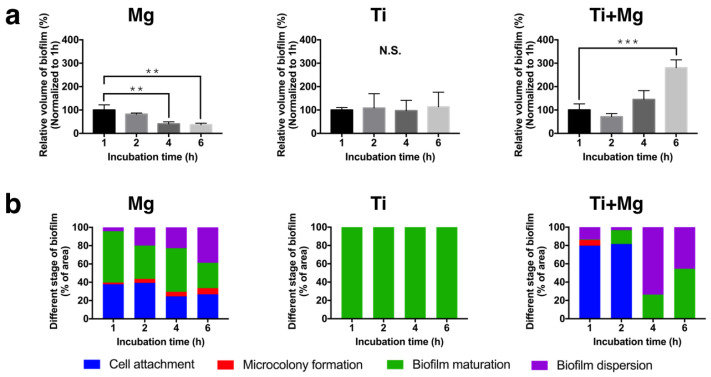
(**a**) The volumetric change for the Mg, Ti, and Ti^+Mg^ groups, and (**b**). the percentage of biofilms after different period of incubations (*n* = 3 per group; N.S. no significant difference, ** *p* < 0.01, and *** *p* < 0.005).

Figure 5a shows the normalized volume of biofilms, which were stained by crystal violet staining; the numbers were normalized, respectively, to the corresponding volumes of biofilm in all stages at one hour of incubation in each group. Normalization is adopted, as we aim to analyze how the bacterial colonization varies further in the three groups once the biofilm has developed. After six hours of incubation, the volume of biofilm significantly decreased to 44.7% for the Mg group, slightly increased to more than 100% for the Ti group, and greatly increased to 280.9% for the Ti^+Mg^ group. The initial formation of the biofilms in the Ti^+Mg^ group grew very slowly. This may be restrained by the alkaline environment, due to Mg ions.

As a reminder, we consider four stages of biofilm formation, including cell attachment (stage 1), microcolony formation (stage 2), biofilm maturation (stage 3), and biofilm dispersion (stage 4). We also recorded the volumetric percentage of biofilm in different stages (calculated by the aforementioned AI system), as shown in Figure 5b. For the Mg group, the percentage of attached cells was approximately 40% at one hour after incubation, and decreased to 26.8% at six hours after incubation. The percentage of mature biofilm decreased from 56.2% after one hour to 27.8% after six hours of incubation. The microcolony formation stage accounted for only a small percentage all the time. The percentage of dispersed bacteria increased, which is a sign of antibacterial effect, since the dispersed planktonic bacteria can be more easily eradicated in the alkaline environment. As for the Ti group, biofilm almost all developed to maturity during the 1–6 h of incubation. It implies that once bacterial infection has developed on the surface of Ti, it is highly unlikely to be eradicated. While the Mg group and Ti group both developed mature biofilms within one hour of incubation, for the Ti^+Mg^ group, the mature biofilm did not appear until after two hours of immersion, indicating that the development of biofilms on the Ti substrate may be delayed by the Mg ion-induced alkaline environment. After the mature biofilm appeared in the Ti^+Mg^ group, the mature biofilms increased from 26.4% at four hours to 54.6% at six hours after incubation, respectively.

Both the colonization of biofilms in the Ti group and Ti^+Mg^ group indicates that Ti, which is resistant to initial bacterial attachment, may offer a suitable matrix for maturely formed biofilms to expand.

### 3.6. Viability of Planktonic Bacteria

**Figure 6:**
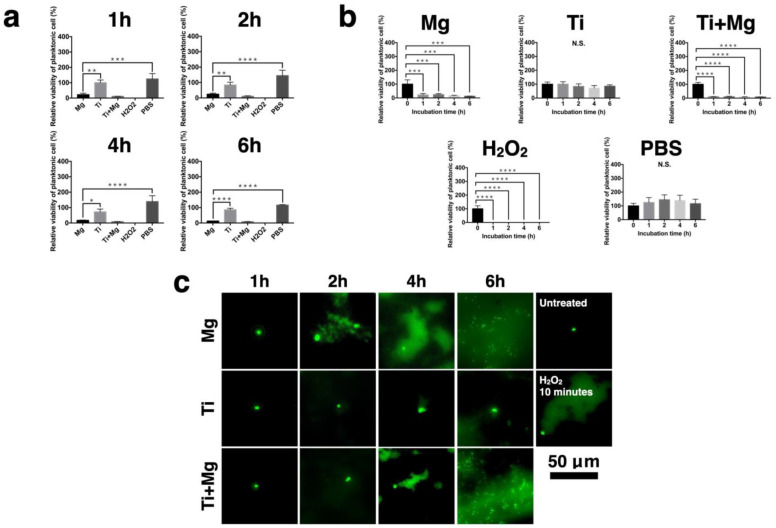
The viability of planktonic bacterial cell. (**a**) Bacterial cell viability qualification by MTT assay with respect to different periods of incubation; (**b**) bacterial cell viability qualification by MTT assay with respect to different groups; (**c**) comet assay of planktonic bacteria reflects the degree of DNA damage. (*n* = 3 per group; N.S. no significant difference, * *p* < 0.05, ** *p* < 0.01, *** *p* < 0.005, and **** *p* < 0.001).

The viability of planktonic bacteria was evaluated and normalized for the Mg, Ti, Ti^+Mg^, PBS, and 10% H_2_O_2_ groups, where 10% H_2_O_2_ is the positive control and PBS is the negative control group, and is presented in Figure 6a,b. As we can see, the Mg and Ti^+Mg^ groups showed a similar profile to the H_2_O_2_ group, whereas the Ti group was similar to the PBS group. Therefore, Mg and Ti^+Mg^ have lower bacteria viability than the Ti group.

### 3.7. DNA Damage of Bacteria in Different Environments

The DNA damage of planktonic DH5-alpha *E. coli* cultured for the Mg, Ti, and Ti^+Mg^ group were qualified by comet assay. For comparison, we also have the untreated group (negative control) and the H_2_O_2_ treated group (10 min, positive control). It can be seen from Figure 6c, captured by fluorescence microscopy, that for the untreated group (negative control), after electrophoresis, there is a clear nucleus without any halo ring or smoke-like matter, implying that the bacterial cells are live. In contrast, for the H_2_O_2_ treated group, there is a visible comet tail and smoke-like matter, which is evidence of DNA damage. The direction of the tail always pointed to the upper right-hand side from the (broken) nuclei due to the employed electrophoresis. As we know, the longer the comet tails appear, the more intense damage had been inflicted on DNA [60]. After one hour of incubation, the Mg and Ti^+Mg^ groups presented slight smoke-like halo rings, meaning that the cell had been slightly disrupted. The visible comet tail appeared in the Mg group after two hours of incubation. Up until six hours, the Mg and Ti^+Mg^ groups both showed not only large comet tails and smoke-like matter, but also fragments of nuclei. Cell apoptosis of DH5-alpha *E. coli* is characterized by the size of DNA fragmentation ranging within 50–1000 nm, given the size of its nucleosome oligomers [61,62]. The size of small fragments of the Mg and Ti^+Mg^ group after six hours of incubation was approximately 1000 nm, confirming that the cell had been destroyed. On the other hand, the Ti group did not show any comet tails after six hours of incubation, indicating that the cell was intact.

## 4. Discussion

The main aim of this study was to evidence that (1) an alkaline environment is harmless to adherent bacteria covered by well-formed biofilms, and (2) the spalling mechanism of the biodegradable Mg surface can disrupt the structure of biofilm to release the bacteria into the planktonic phase, which can be more easily eradicated via the alkaline environment induced by Mg ions. To this end, we studied the morphology of biofilm formation respectively for the Ti, Mg, and Ti^+Mg^ groups. The observation can be summarized as below:

The difference between the Ti and the Ti^+Mg^ group is that Ti^+Mg^ was soaked in an alkaline environment with Mg ions released from the accompanied Mg, whereas Ti stayed in a neutral environment. Figure 3c shows that for the Ti^+Mg^ group, the pH value of bacterial culture media was increased up to around 9 within one hour of incubation, and continuously maintained as such until after six hours of incubation; for the Ti group, the pH value was always around 7. Taking the volumes of biofilms after one hour of incubation as benchmarks, Figure 5a shows that the formation of biofilms for the Ti and Ti^+Mg^ group followed the same trend, i.e., the volumes of the biofilms both generally increased with time. Figure 5a,b show that for the Ti group, among the slightly expanding biofilms, almost all were maturely formed in stage 3, indicating steady growth of biofilms. Many studies have reported that a high pH value provides unfriendly environment to planktonic bacteria [30,38,39], which explained that in Figure 5a,b the alkaline environment successfully delayed the development of biofilms on the substrate of the Ti^+Mg^ group for about 2 h of incubation. In particular, during this period, not only did the volume of biofilms decrease, but also the immature biofilms (stage 1) became dominating. Nevertheless, between one hour and two hours of incubation, planktonic bacteria overcame the challenge of adverse attachment to form mature biofilms on the surface of Ti^+Mg^; the bacteria colonization volume-wisely expanded until six hours of incubation, and the percentage of mature biofilms (stage 3) also increased. Notably, after four hours of incubation, there was barely cell attachment and microcolony formation in the Ti^+Mg^ group, implying the increasing volume of biofilms in all stages until six hours of incubation may have been an expansion of the mature biofilms instead of a result of newly formed mature biofilms. All of the previous observations indicate that once mature biofilm has appeared on the surface of Ti^+Mg^, the alkaline environment is harmless to the inherent bacteria, and the further bacterial colonization is unpreventable. This may explain that clinically bacterial infection leads rapidly to a dangerous situation, since the medical treatment mainly generates an unfriendly environment for bacteria, which may be ineffective to the adherent bacteria covered by mature biofilms.

The effect of spalling mechanism can be demonstrated by comparing the Mg group and Ti^+Mg^ group, as they both have alkaline environments. The volume of biofilms for the Mg and the Ti^+Mg^ group both decreased slightly during the first two hours of immersion, which may be an effect of the alkaline environment (Figure 5a). Between these two groups, there is another physical difference—the relatively hydrophilic Mg has a weaker resistance to cell attachment than the hydrophobic Ti, which was confirmed by Figure 4b (one hour of incubation). As revealed by Figure 5b, within one hour of immersion, the Mg group has grown mature biofilms, yet in the Ti^+Mg^ group, the biofilm only slightly reached maturity before two hours of incubation. The development of biofilms in Ti^+Mg^ group was much slower. We further observed that after the bacteria have conquered the adverse attachment in the Ti^+Mg^ group after two hours of immersion to develop mature biofilm, the rapid extension of biofilms and increasingly dominating mature biofilm (stage 3) supported each other, and therefore the colonization of biofilms was unstoppable even in an alkaline environment. On the contrary, the spalling mechanism of degradable Mg continuously disrupted the biofilms, including the mature ones, which is evidenced by Figure 4c, as the biofilms near the crack of the Mg surface were broken. As a result, for the Mg group, the damaged biofilms released the attached bacteria to the antibacterial alkaline broth so that the percentage of mature biofilms followed a decreasing trend, leading to a continuously shrinking territory of biofilms.

The difference of the Mg group and the Ti group could be a combination of the previous two items. Basically, the Mg group has an alkaline environment to delay the initial development of biofilms, and the surface of the Ti is antibacterial for initial cell attachment. It turned out that an alkaline environment is less harmful to the initial development of biofilms, which was evidenced by Figure 4b, as the Mg group has much more biofilm after one hour of incubation than the Ti group. Taking the corresponding volumes of biofilms at one hour of incubation as benchmarks, Figure 5a showed that after six hours of incubation, the volumes corresponding to Mg group had shrunk to 44.7%, whereas that of the Ti group grew up to more than 100%. Moreover, in Figure 5b, the Mg group showed the percentage of mature biofilms among all biofilms decreased from 55% after one hour of incubation to around 40% after six hours of incubation; in contrast, the bacteria attached on the surface of Ti had all rapidly developed to mature biofilm (almost 100%) within one hour of incubation. In summary, the stable surface of nondegradable Ti has stronger resistance to bacterial attachment than Mg. However, if mature biofilm has formed, the spalling mechanism of degradable Mg is able to break the well-formed biofilm, weakening the occupation of biofilms, whereas the Ti offers a stable matrix for the further bacterial colonization.

All experimental results were obtained merely within six hours of immersion. From these data, we have seen significant effects of the degradable Mg on disturbing the growth of biofilms, which is compared to the Ti and Ti^+Mg^ group. The degradation of Mg can continue for much longer than six hours, and may further weaken bacterial infection. The potential of degradable metal to be modified as a feasible implant material was proposed by this study.

## 5. Conclusions

This study showed that the mature biofilm can protect the covered bacteria from the outside antibacterial (alkaline) environment. The widely used Ti has outstanding antibacterial property, so much so that the planktonic bacteria have difficulty to attach on it. Nevertheless, it is almost unlikely to get rid of a mature biofilm once it has successfully developed on the surface of Ti. On the other hand, degradable Mg, which has a rather weak resistance to bacterial attachment, can disrupt the biofilms by its spalling mechanism, which continues until Mg is completely degraded. The released bacteria from the broken biofilms were hardly viable due to the Mg ion-induced alkaline environment. Such a two-step mechanism continues to weaken the bacterial colonization. This study implies that degradable materials may be feasible implants, since they have the potential to defeat slight infection after a surgery, which is of clinical relevance.

## Figures and Tables

**Table 1:** Summary of training and testing datasets for biofilm features.

	Train	Test	Total
Mg	630	69	699
Ti	2541	282	2823
Ti^+Mg^	890	98	988

**Table 2:** The surface composition of Mg and Ti substrates (*n* = 5 per group).

Material	Immersion Time (h)	Na	Mg	Al	Si	P	Cl	K	Ti
Mg	0	0	100 ± 0	0	0	0	0	0	0
	1	2.08 ± 1.09	94.38 ± 4.13	0.01 ± 0.02	0.05 ± 0.09	1.08 ± 0.94	2.32 ± 2.61	0.08 ± 0.14	0
	2	0.84 ± 0.90	95.36 ± 0.60	0	0	3.80 ± 0.90	0	0	0
	4	0.92 ± 0.88	91.51 ± 2.26	0	0	6.93 ± 0.94	0.65 ± 1.12	0	0
	6	0	97.22 ± 0.38	0	0	2.78 ± 0.38	0	0	0
Ti	0	0	0	0	0	0	0	0	100 ± 0
	1	0	0	0	0	0	0	0	100 ± 0
	2	0	0	0	0	0	0	0	100 ± 0
	4	0	0	0	0	0	0	0	100 ± 0
	6	0	0	0	0	0	0	0	100 ± 0
Ti (Ti^+Mg^ group)	0	0	0	0	0	0	0	0	100 ± 0
	1	0	0	0	0	0	0	0	100 ± 0
	2	0	0	0	0	0	0	0	100 ± 0
	4	0	0	0	0	0	0	0	100 ± 0
	6	0	0	0	0	0	0	0	100 ± 0

**Table 3:** Confusion matrix for biofilm classification using the proposed CNN model for Mg, Ti and Ti^+Mg^ groups, respectively.

MG Group		Detected Results				
		BM	Stage 1	Stage 2	Stage 3	Stage 4
**Actual Label**	BM	9	0	0	1	1
	Stage 1	2	19	1	0	0
	Stage 2	0	0	13	0	1
	Stage 3	1	0	0	10	2
	Stage 4	0	0	2	0	7
	ACC (%)	75	100	81	91	64
	Total	12	19	16	11	11
**Ti Group**		**Detected Results**				
		BM	Stage 1	Stage 2	Stage 3	Stage 4
**Actual Label**	BM	32	4	0	3	0
	Stage 1	10	24	0	0	1
	Stage 2	0	0	0	0	0
	Stage 3	21	4	0	129	8
	Stage 4	1	1	0	16	28
	ACC (%)	50	73	0	87	76
	Total	64	33	0	148	37
**Ti^+Mg^ Group**		**Detected Results**				
		BM	stage 1	stage 2	stage 3	stage 4
**Actual Label**	BM	12	0	0	1	1
	stage 1	1	10	0	5	0
	stage 2	0	0	0	0	0
	stage 3	1	2	0	24	0
	stage 4	0	1	0	0	40
	ACC (%)	86	77	0	80	98
	Total	14	13	0	30	41

BM: base material; ACC: accuracy.

## Data Availability

The data presented in this study are available on request from the corresponding author. The data are not publicly available since they are raw data.
